# Novel Boronate Probe Based on 3-Benzothiazol-2-yl-7-hydroxy-chromen-2-one for the Detection of Peroxynitrite and Hypochlorite

**DOI:** 10.3390/molecules26195940

**Published:** 2021-09-30

**Authors:** Julia Modrzejewska, Marcin Szala, Aleksandra Grzelakowska, Małgorzata Zakłos-Szyda, Jacek Zielonka, Radosław Podsiadły

**Affiliations:** 1 Institute of Polymer and Dye Technology, Faculty of Chemistry, Lodz University of Technology, Stefanowskiego 12/16, 90-924 Lodz, Poland; julia.modrzejewska@dokt.p.lodz.pl (J.M.); marcin.szala@p.lodz.pl (M.S.); aleksandra.grzelakowska@p.lodz.pl (A.G.); 2 Institute of Molecular and Industrial Biotechnology, Faculty of Biotechnology and Food Sciences, Lodz University of Technology, Stefanowskiego 2/22, 90-537 Lodz, Poland; malgorzata.zaklos-szyda@p.lodz.pl; 3 Department of Biophysics, Medical College of Wisconsin, 8701 Watertown Plank Road, Milwaukee, WI 53226, USA

**Keywords:** fluorescent probe, coumarin derivatives, peroxynitrite

## Abstract

Derivatives of coumarin, containing oxidant-sensitive boronate group, were recently developed for fluorescent detection of inflammatory oxidants. Here, we report the synthesis and the characterization of 3-(2-benzothiazolyl)-7-coumarin boronic acid pinacol ester (**BC-BE**) as a fluorescent probe for the detection of peroxynitrite (ONOO^–^), with high stability and a fast response time. The **BC-BE** probe hydrolyzes in phosphate buffer to 3-(2-benzothiazolyl)-7-coumarin boronic acid (**BC-BA**) which is stable in the solution even after a prolonged incubation time (24 h). **BC-BA** is slowly oxidized by H_2_O_2_ to form the phenolic product, 3-benzothiazol-2-yl-7-hydroxy-chromen-2-one (**BC-OH**). On the other hand, the **BC-BA** probe reacts rapidly with ONOO^−^. The ability of the **BC-BA** probe to detect ONOO^–^ was measured using both authentic ONOO^–^ and the system co-generating steady-state fluxes of O_2_^•^^–^ and ^•^NO. **BC-BA** is oxidized by ONOO^–^ to **BC-OH**. However, in this reaction 3-benzothiazol-2-yl-chromen-2-one (**BC-H**) is formed in the minor pathway, as a peroxynitrite-specific product. **BC-OH** is also formed in the reaction of **BC-BA** with HOCl, and subsequent reaction of **BC-OH** with HOCl leads to the formation of a chlorinated phenolic product, which could be used as a specific product for HOCl. We conclude that **BC-BA** shows potential as an improved fluorescent probe for the detection of peroxynitrite and hypochlorite in biological settings. Complementation of the fluorescence measurements by HPLC-based identification of oxidant-specific products will help to identify the oxidants detected.

## 1. Introduction

Coumarin skeleton is frequently used to construct a range of fluorescent dyes due to high fluorescent quantum yields and tunable emission wavelengths. The emission of coumarin-based fluorophores can be finely tuned by appropriate substitution in 2*H*-chromen-2-one skeleton. Fluorescence can be red shifted by the placement of electron-donating groups in the six- or seven-position or electron-accepting groups in the three- or four-position of the skeleton [1].

A widely used example of coumarin dyes is C.I. Disperse Yellow 82 [2] which, in the 7-position of the coumarin ring, contains the *N*,*N*-diethylamino group and, in the 3-position, a benzimidazole residue. Derivatives of coumarin generally show good photostability which is rather unusual among fluorescent dyes [3].

7-Hydroxy-2*H*-chromen-2-one (also known as 7-hydroxycoumarin, **COH**, or umbelliferone) is widely used as a fluorophore in sensors and probes due to the relatively simple methods of masking its hydroxyl group and a high water solubility (621 mg L^−1^ [4]). Umbelliferone was chosen as a scaffold to construct a wide variety of probes, by listing only several example, e.g., for the detection of anions [1,5,6], formaldehyde [7], hydrogen sulfide [8,9,10], biothiols [11,12], enzymes [13,14], Ser/Thr protein phosphatases [15], sulfane sulfurs [16], peroxynitrite [17], and hydrogen peroxide [18,19].

However, **COH** has several disadvantages, including a short wavelength UV-absorption at 320 nm, a relatively low extinction coefficient (ε = 1.3 × 10^4^ M^−1^ cm^−1^) [20], and a pH dependent fluorescence efficiency [17,20,21]. Therefore, especially for biomedical applications, alternative coumarin analogs with red-shifted absorption are sought [22]. 

The detection of reactive oxygen and nitrogen species is becoming more important these days. An excess of reactive oxygen species (ROS) and reactive nitrogen species (RNS) are implicated in pathologies such as cancer, cardiovascular, and neurodegenerative diseases [23]. To understand how these oxidative stressors participate in cellular function it is important to detect when, where, and what kind of specific products are produced. Significant information about reactive oxidants can be obtained using high performance liquid chromatography, mass spectrometry or other analytical procedure, but only connecting these methods with fluorescence and chemiluminescence approaches provide real-time monitoring.

There are two main fluorescence and chemiluminescence strategies for ROS/RNS detection. The first one is based on aromatic compounds that undergo oxidation to a fluorescent product (redox probes) and, the second, in which compound contains masked fluorophore. These are often called “non-redox” probes, as the fluorescence of the probe is uncovered through nucleophilic attack of the reactive species on the blocking group. Boronates are most often investigated as leaving groups [24,25,26,27,28,29,30,31].

Continuing our research on the synthesis of low molecular weight boronate probes for the detection of peroxynitrite [27,28,29], we focused our efforts on the synthesis of a coumarin probe with the excitation band located above 400 nm. With the exception of coumarin 7-boronic acid, **CBA**, so far boronate probes based on the coumarin skeleton have been obtained by the boronobenzylation process [30]. However, the oxidative conversion of boronobenzyloxycarbonyl- and boronobenzylcoumarin derivatives not only produces a fluorophore but also releases quinone methide (QM) moiety. The self-immolation of such a moiety results in the delayed formation of a fluorescent product, while fluorophores with direct derivatization by the boronate group produce the fluorescent product instantly upon oxidation [28]. Another potential disadvantage of boronobenzylated probes arises from the fact that QM as an electrophile may influence the redox state of the cell and thus influence the redox environment studied. [31]

**Scheme 1:**
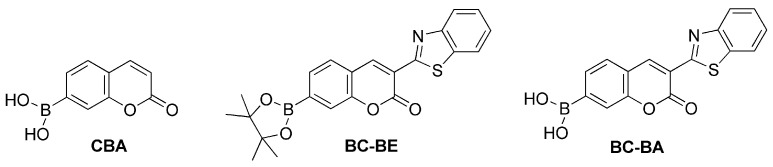
Pro-coumarin boronate based probes.

Here, we report the synthesis and the characterization of a novel 3-(2-benzothiazolyl)-7-coumarin boronic acid pinacol ester, **BC-BE**, an analogue of the **CBA** probe with a benzothiazole residue in the 3-position of the coumarin ring (Scheme 1). In aqueous solutions containing a phosphate buffer (pH 7.4), the **BC-BE** probe undergoes fast hydrolysis to its boronic acid (**BC-BA**). Oxidation of **BC-BE** results in instantaneous formation of 3-benzothiazol-2-yl-7-hydroxy-chromen-2-one **BC-OH**, a fluorescent dye with improved photophysical properties as compared to **COH**.

## 2. Results

### 2.1. Synthesis

**Scheme 2:**
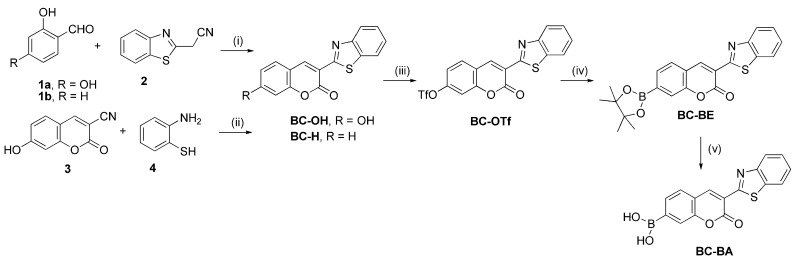
Synthesis of novel boronates based on 3-benzothiazol-2-yl-7-hydroxy-chromen-2-one. Reagents and conditions: (**i**) 1. EtOH, piperidine, rt, 3 h; 2. 10% HCl, reflux, 3 h; (**ii**) acetic acid, 150 °C, MW, 40 min; (**iii**) PhN(SO_2_CF_3_)_2_, DCM, TEA, reflux, 4 h; (**iv**) Pd(dppf)Cl_2_, dppf, AcOK, 1,4-dioxane, 100 °C, MW, 45 min; (**v**) phosphate buffer, pH 7.4, 30 min.

The **BC-BE** boronate probe was obtained in a two-step protocol starting from the fluorophore **BC-OH**, prepared from 3-cyano-7-hydroxycoumarin (**3**) and *ortho*-aminothiophenol (**4**) according to the published procedure [32] (Scheme 2). In the first step, the phenolic hydroxyl group was converted into the appropriate triflate **BC-OTf** with a 99% yield. In the second step, **BC-OTf** was transformed to **BC-BE** in a Pd(dppf)Cl_2_-assisted reaction with bis(pinacolato)diboron. Since the probe was designed to detect peroxynitrite, the anticipated minor product, **BC-H** coumarin derivative was also prepared via a classical one-step condensation between appropriate *ortho*-hydroxybenzaldehyde (**1b**) and benzothiazole-2-carbonitrile (**2**).

### 2.2. Spectroscopic Response of ***BC-BE***

**Figure 1:**
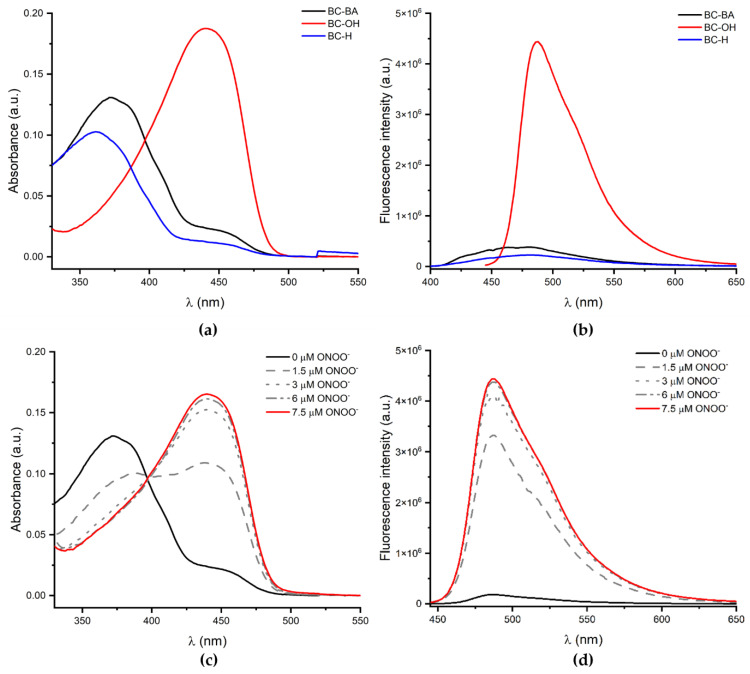
Absorption and emission spectra of studied coumarins recorded in mixture of phosphate buffer (0.1 M, pH 7.4) and EtOH (10%). (**a**) Absorption spectra of **BC-OH**, **BC-BA,** and **BC-H** (5 µM each); (**b**) fluorescence spectra of **BC-OH**, **BC-BA**, and **BC-H** (5 µM each, λ_ex_ = λ_max_); (**c**) absorption spectra of **BC-BA** probe (5 µM) recorded before and after bolus addition of different amount of peroxynitrite (0, 1.5, 4.5, 6, 7.5 µM); (**d**) emission spectra of the **BC-BA** probe (5 µM, λ_ex_ = 442 nm) recorded before and after bolus addition of different amount of peroxynitrite (0, 1.5, 4.5, 6, 7.5 µM).

Introduction of the benzothiazolyl group into the coumarin skeleton has only a minor effect on the acid-base properties of the phenolic hydroxyl group. In fact, the reported pKa values of **COH** and **BC-OH** are 6.89 [33] and 7.0 [34], respectively. Photophysical properties of the novel boronate probe and the parent hydroxycoumarin **BC-OH**, as well as **COH** are compared in Table 1. Figure 1 shows absorption and emission spectra of **BC-BA**, **BC-H** and **BC-OH**. Presented data demonstrate that boronate group significantly reduces the emission of the **BC-OH** coumarin. **BC-BA** exhibited a maximal UV-absorption at 371 nm (ε = 25,500 M^−1^ cm^−1^) and a maximal emission at 473 nm (Φ = 0.17). **BC-OH** has absorption and emission bands red shifted in comparison with **BC-BA**. More importantly, this dye has a higher quantum yield of emission than **BC-BA** and a higher extinction coefficient (resulting in higher brightness) than **COH**. Bolus addition of peroxynitrite to the solution of the **BC-BA** probe red shifts the absorption band (Figure 1c) and turns on fluorescence (Figure 1d).

### 2.3. Reactivity towards Biological Oxidants

**Figure 2:**
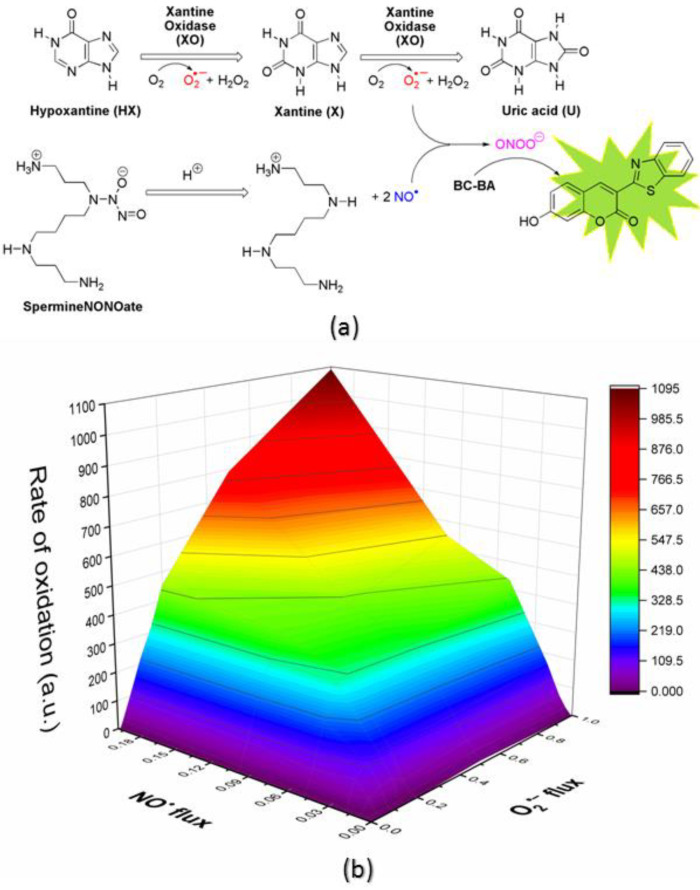
Measurement of peroxynitrite formation in situ by monitoring oxidation of **BC-BA** (20 µM) using a 96-well fluorescence plate reader. (**a**) The formation of O_2_^•–^ from incubations containing hypoxanthine HX (100 µM) and xanthine oxidase XO (0–0.4 milliunits/mL) in a phosphate buffer (pH 7.4; 50 mM) containing DTPA (100 µM), and ^•^NO generation from a thermal decomposition of spermine-NONOate (0–50 µM). Peroxynitrite, formed from co-generated fluxes of O_2_^•–^ and ^•^NO, oxidizes **BC-BA** to fluorescent **BC-OH**; (**b**) the rate of increase fluorescence intensity during the oxidation of the **BC-BA** probe in the presence of varying fluxes of O_2_^•–^ and ^•^NO in the phosphate buffer (pH 7.4, 50 mM) with DTPA (100 µM), HX/XO, spermine-NONOate, and CAT (100 U/mL). The fluorescence intensity (λ_ex_ at 405 nm, λ_em_ at 535 nm) of the reaction mixtures consisting of **BC-BA** (20 µM) and HX/XO (O_2_^•–^ flux: 0–1 µM/min) and spermine-NONOate (^•^NO flux: 0–0.2 µM/min) was measured over a period of 2 h.

The effectiveness of the **BC-BA** probe for the detection of peroxynitrite was also measured using the system co-generating steady fluxes of O_2_^•–^ (from hypoxanthine (HX) and xanthine oxidase XO) and ^•^NO (from spermine-NONOate). The profile of the **BC-BA** probe oxidation as a measure of ONOO^–^ formation in a matrix of various fluxes of O_2_^•–^ and ^•^NO is shown in Figure 2a.

**Figure 3:**
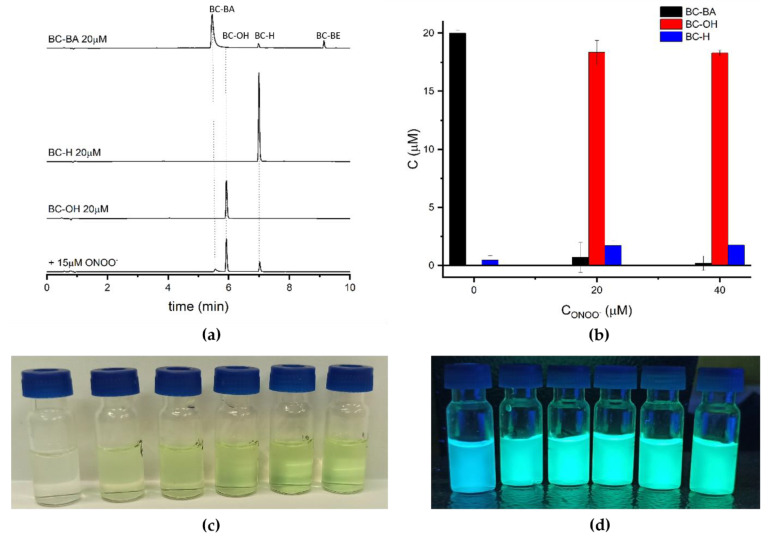
Peroxynitrite-induced oxidation of the **BC-BA** probe. (**a**) HPLC traces of the mixture of **BC-BA**, **BC-OH**, and **BC-H** (20 µM each) and the reaction mixtures of **BC-BA** (20 µM) with peroxynitrite (15 µM) after 5 min of incubation. Tests were conducted in aqueous solutions containing EtOH (10%), phosphate buffer (0.1 M, pH 7.4) and dtpa (10 µM). The traces were collected using an absorption detector set at 330 nm; (**b**) HPLC based titration of **BC-BA** probe (20 µM) with ONOO^–^ (0–40 µM). Pictures of **BC-BA** probe (20 µM) with different amount of peroxynitrite (0, 6, 12, 18, 24, 30 µM): (**c**) in day light; (**d**) under UV.

**Scheme 3:**
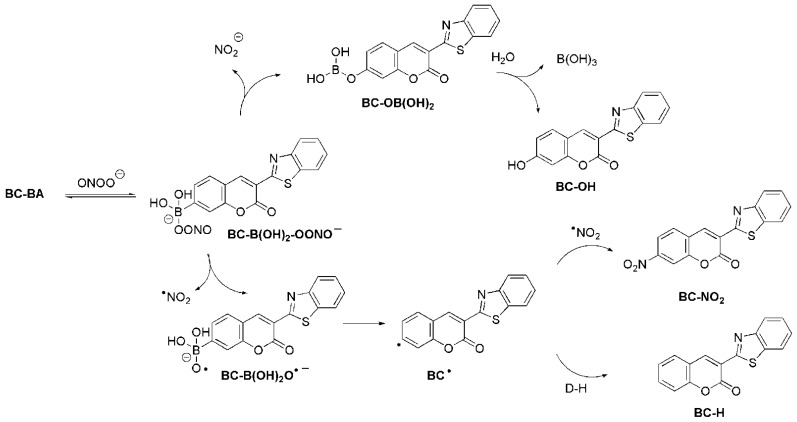
Peroxynitrite induced oxidative conversion of **BC-BA** leading to benzothiazolyl-coumarin derived dyes **BC-OH**, **BC-H,** and **BC-NO_2_**.

Next, we determined the stoichiometry of the reaction between **BC-BA** and peroxynitrite. Using HPLC, we analyzed products formed after the bolus addition of ONOO^–^ to the phosphate buffer containing the **BC-BA** probe. Figure 3a shows that, after 5 min of incubation, we observed the formation of the **BC-OH** coumarin as the main product. The formation of **BC-OH** can be observed with the naked eye, as shown in Figure 3c. The hue of fluorescence also changes (Figure 3d). Figure 3b showed that **BC-BA** reacts with ONOO^–^ forming the **BC-OH** dye with c.a. 90% yield. These results are consistent with a previously reported reactivity of arylboronate-derived probes toward peroxynitrite [30]. Peroxynitrite-induced oxidation of boronic acid Ar-B(OH)_2_ or ester Ar-B(pin) proceeds via two pathways, and typically leads to the formation of minor but ONOO^–^-specific products (ArNO_2_, ArH) in addition to the major phenolic product (ArOH). Therefore, we anticipated that the **BC-BA** reaction with ONOO^–^ would produce the corresponding minor products (**BC-NO_2_** and **BC-H**), as shown in Scheme 3. However, under the experimental conditions used we only detected **BC-H** (c.a. 9% yield), probably due to the usage of ethanol as an organic co-solvent, which efficiently reduces the phenyl-type radical **BC^•^** to **BC-H** (Scheme 2). Previously, it has shown that in the presence of phenyl radical scavengers (2-propanol), the phenyl radical formed in the radical pathway is almost quantitatively converted into the product in which the boronate moiety is replaced by a hydrogen atom [36,37].

**Figure 4:**
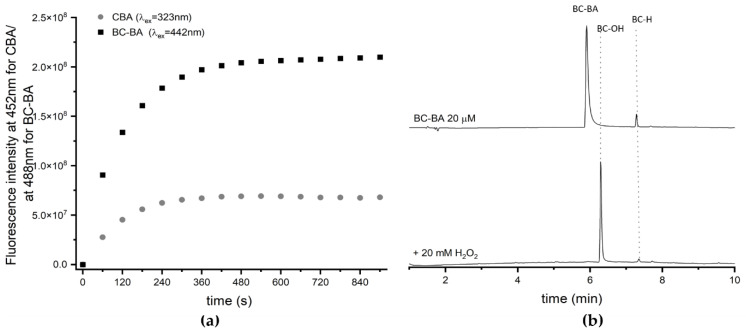
(**a**) Kinetic profiles of product formation during the oxidation of probes **BC-BA** and **CBA** (5 µM each) by H_2_O_2_ (5 mM). Experiments were repeated three times independently. Representative result displayed; (**b**) HPLC traces of the **BC-BA** probe (20 µM) before and after addition of H_2_O_2_ (20 mM).

Boronate probe is also oxidized by other inflammatory oxidants such as hydrogen peroxide and hypochlorous acid [27,28,29,30]. Therefore, we also tested the reactivity of **BC-BA** toward H_2_O_2_ and HOCl. We also compared the kinetic profile of **BC-OH** formation during the oxidation of **BC-BA** to the profile of the **COH** formation from a simple boronate probe, coumarin-7-boronic acid (**CBA**). Figure 4a shows the buildup of emission at 442 nm during the reaction between those boronic probes and H_2_O_2_ (5 mM). It is evident that both probes release products (**BC-OH** or **COH**) with comparable reaction rates. However, the signal intensity of **BC-OH** is 3-fold higher than the signal emission of **COH**, demonstrating significantly higher brightness of the product, consistent with the fluorescence parameters listed in Table 1. It is also worth emphasizing that in the reaction of the **BC-BA** probe with H_2_O_2_, the **BC-OH** coumarin is formed as the sole product (Figure 4b). [27,28,29,30].

**Figure 5:**
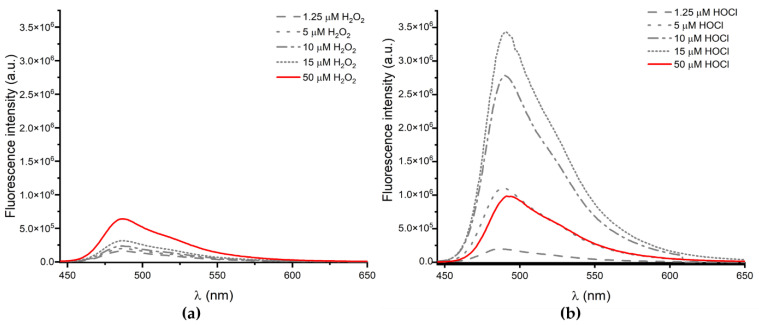
Emission spectra of the **BC-BE** probe (5 µM, λ_ex_ = 442 nm) recorded before and after bolus addition of different amount (0, 1.5, 4.5, 6, 7.5 µM) of: (**a**) H_2_O_2_ (after 24 h of incubation); (**b**) HOCl (after 30 min of incubation). Tests were conducted in aqueous solutions containing EtOH (10%), phosphate buffer (0.1 M, pH 7.4) and dtpa (10 µM).

**Figure 6:**
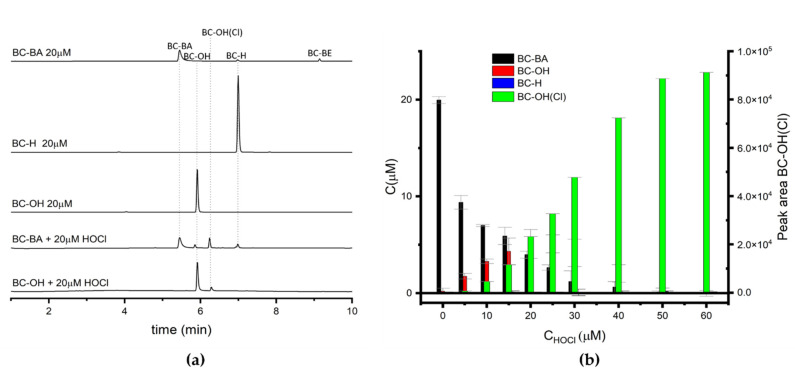

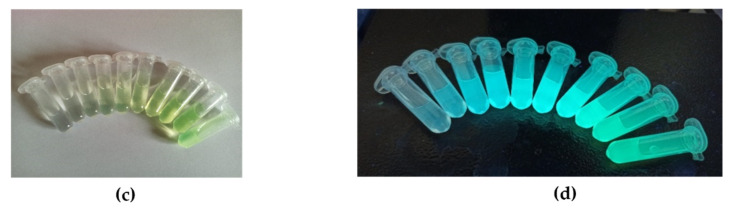
HOCl-induced oxidation of the **BC-BA** probe. (**a**) HPLC traces of the mixture of **BC-BA**, **BC-OH** and **BC-H** (20 µM each) and the reaction mixtures of **BC-BA** (20 µM) and **BC-OH** (20 µM) with HOCl (20 µM) after 15 min of incubation. Tests were conducted in aqueous solutions containing EtOH (10%), phosphate buffer (0.1 M, pH 7.4) and dtpa (10 µM). The traces were collected using an absorption detector set at 330 nm; (**b**) HPLC based titration of the **BC-BA** probe (20 µM) with HOCl (0–60 µM). Pictures of the **BC-BE** probe (20 µM) with different amount of HOCl (0–60 µM): (**c**) in day light; (**d**) under UV.

Emission spectra recorded after the addition of a micromolar concentration of H_2_O_2_ into the phosphate buffer solution containing **BC-BA** are shown in Figure 5a. The intensity of recorded spectra are lower in comparison with the intensity of fluorescence measured after the reaction of **BC-BA** with ONOO^–^ (Figure 1d). For comparison, we also studied the reaction of **BC-BA** with hypochlorous acid. Emission spectra (Figure 5b) reveal that the **BC-BA** probe is converted to the **BC-OH** dye. In contrast to the reaction of the probe with hydrogen peroxide, however, the signal intensity is significantly higher. HPLC analysis of the reaction mixture (Figure 6a) shows that besides **BC-OH**, another product is also formed. We attributed this product to the chlorinated derivative of the coumarin BC-OH(Cl) since the product with the same retention time (6.1 min) was also detected after the addition of HOCl to the **BC-OH** solution. By analyzing the disappearance of the **BC-BA** probe and the concentration of products formed with an increase in the amount of added HOCl, it can be seen that the maximum yield of **BC-OH** reaches only ca. 25%. Moreover, an excess of HOCl causes the disappearance of **BC-OH**. This is consistent with our previous investigations in which we demonstrated that the phenolic product (luciferin or 6-(2-benzothiazolyl)-2-naphthalenol) released from the boronate probes (LBA, PCL-1 or NAB-BE) undergoes a further reaction with HOCl, leading to the formation of 7′-chloroluciferin [27,28] or 6-(1,3-benzothiazol-2-yl)-1-chloronaphthalen-2-ol [29], a product specific for HOCl.

### 2.4. The Effect of Compounds on Cell Metabolic Activity

**Figure 7:**
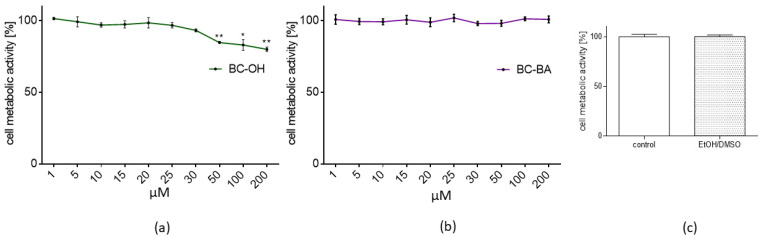

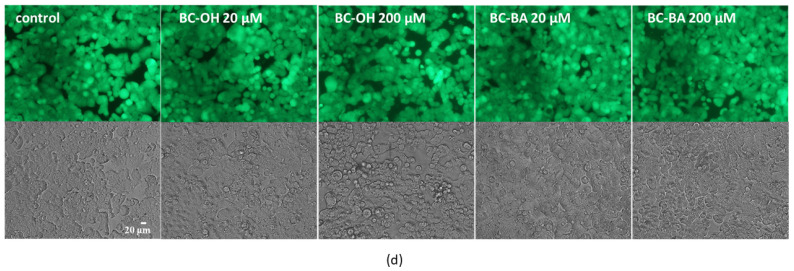
The influence of **BC-OH** (**a**) and **BC-BA** (**b**) on HT29 cell metabolic activity, determined by MTT assay after 8 h exposure; control cells were not exposed to any compound but the vehicle (EtOH/DMSO (9:1) (**c**); values are means ± SEM, *n* ≥ 7; statistical significance was calculated versus control cells (untreated), * *p* ≤ 0.05, ** *p* ≤ 0.01. Morphology of HT29 cells stained with 2 μM calcein AM observed after incubation with 20 and 200 μM of **BC-OH** and **BC-BA** (**d**); randomly chosen fields were photographed at × 200 with fluorescent microscope Nikon TS Eclipse 100.

To determine the potential usage of synthesized compounds in cell-based assays, next the impact of probes on the metabolic activity of human colon cancer HT29 cells was studied. Compounds were dissolved in ethanol/dimethylsulfoxide mixture (9:1) and added to the cells in a volume not exceeding 2% of the medium, which had no cytotoxic effect on cells. To determine the cytotoxic potential of studied compounds, cells were incubated for 8 h in their presence at the range of 0–200 µM concentration. As it is presented in Figure 7a–c, compounds **BC-OH** and **BC-BA** at 20 µM concentration had no impact on HT29 cells’ metabolic activity; therefore, both of them can be potentially used in studies requiring cell incubation for at least 20 min. The direct comparison of cytotoxic potential within the studied range of concentration revealed that compound **BC-OH** was more cytotoxic than **BC-BA**. The highest cytotoxicity of **BC-OH** was detected for 200 µM with decreased metabolic activity by almost 20% compared to the control cells. Microscopic observations performed with calcein AM ester confirmed the lack of cytotoxic effects of both compounds at 20 µM on HT29 cells, as shown in Figure 7d. In healthy cells with active esterases, there is a visible strong cytosolic green fluorescence of calcein. Cells incubated with 200 µM of **BC-OH** had lower cytoplasmic esterase activity, thus a decreased green fluorescence of calcein was observed, as well as the presence of some less attached and more rounded cells.

## 3. Materials and Methods

### 3.1. General

The reagents used for the synthesis were commercially available. 3-benzothiazol-2-yl-7-hydroxy-chromen-2-one (**BC-OH**) and 3-benzothiazol-2-yl-chromen-2-one (**BC-H**) were synthesized according to published protocols [32,38].

The purity of the final compounds was tested by HPLC (Shimadzu) equipped with a photodiode array detector and analytical column—Phenomenex Kinetex Core-Shell C18 (100 mm × 4.6 mm; 2.6 μm). The mobile phase was a gradient prepared from acetonitrile with 0.1% of TFA (component A) and water with 0.1% of TFA (component B). The analytes were eluted by an increase of A concentration from 10–100% over 10 min at the flow rate of 1.5 mL/min. The column temperature was set at 30 °C.

^1^H NMR spectra were recorded with a Bruker Avance DPX 250 spectrometer at 250 MHz, respectively (see Supplementary materials). Compounds were dissolved in DMSO-d_6_ and TMS was added as internal reference. Mass spectra [TOF MS (ESI+)] were recorded on a Synapt G2-Si spectrometer (Waters, Milford, MA, USA).

Absorption spectra were recorded on UV-Vis-NIR spectrophotometer Jasco-V670. Steady-state and time-resolved fluorescence spectra were recorded on Edinburgh Analytical Instruments FL900.

**BC-OH:**^1^H NMR (250 MHz, DMSO-*d*_6_): δ 11.06 (bs, 1H), 9.16 (s, 1H), 8.16 (d, *J* = 7.7 Hz, 1H), 8.05 (d, *J* = 8.1 Hz, 1H), 7.92 (d, *J* = 8.6 Hz, 1H), 7.57 (t, *J* = 7.6 Hz, 1H), 7.46 (t, *J* = 7.5 Hz, 1H), 6.93 (dd, *J* = 8.6, 2.2 Hz, 1H), 6.86 (d, *J* = 2.1 Hz, 1H). TOF MS ES+ calcd for C_16_H_10_NO_3_S 296.0381, found 296.0390. m.p. 303–305 °C, lit. 302–304 °C [39].

**BC-H**: ^1^H NMR (250 MHz, DMSO-*d*_6_): δ 9.06 (s, 1H), 8.07 (d, *J* = 8.1, 1H), 7.96 (d, *J* = 8.0, 1H), 7.71 (dd, *J* = 7.7, 1.6 Hz, 1H), 7.6–7.59 (m, 1H), 7.55–7.48 (m, 1H), 7.44–7.33 (m, 3H). TOF MS ES+ calcd for C_16_H_10_NO_2_S 280.0432, found 280.0439. m.p. 213–215 °C, lit. 213–215 °C [40].

### 3.2. Synthesis of 3-(Benzo[d]thiazol-2-yl)-2-oxo-2H-chromen-7-yl Trifluoromethanesulfonate (***BC-OTf***)

3-benzothiazol-2-yl-7-hydroxycoumarin **BC-OH** (148 mg, 0.5 mmol) and *N*-phenyl-bis(trifluoromethanesulfonimide) (214 mg, 0.6 mmol) were dissolved in 10 mL of anhydrous chloroform. Triethylamine (0.36 mL, 2.6 mmol) was added to the mixture and the resulting solution was stirred under reflux for 4 h. After cooling to room temperature, it was diluted with chloroform and washed three times with citric acid (20%, 10 mL), three times with water (10 mL), and three times with saturated solution NaHCO_3_ (10 mL). The organic layer was dried over MgSO_4_ and the solvent was removed by rotary evaporation to give a yellowish solid (211 mg, 99%).

**BC-OTf**: ^1^H NMR (250 MHz, DMSO-*d*_6_): δ 9.26 (s, 1H), 8.26 (d, *J* = 8.8 Hz, 1H), 8.18 (d, *J* = 7.7 Hz, 1H), 8.08 (d, *J* = 7.8 Hz, 1H), 7.90 (d, *J* = 2.4 Hz, 1H), 7.64–7.54 (m, 2H), 7.47 (t, 1H). TOF MS ES+ calcd for C_17_H_9_NO_5_S_2_F_3_ 427.9874, found 427.9876. m.p. 167–170 °C.

### 3.3. Synthesis of 3-(2-Benzothiazolyl)-7-coumarin Boronic Acid Pinacol Ester (***BC-BE***)

3-benzothiazol-2-yl-7-trifluoromethanesulfonate coumarin **BC-OTf** (43 mg, 0.1 mmol), bis(pinacolato)diboron (28 mg, 0.11 mmol), Pd(dppf)Cl_2_ (2.2 mg, 0.003 mmol), dppf (1.7 mg, 0.003 mmol), and potassium acetate (29 mg, 0.3 mmol) were dissolved in 5 mL of anhydrous 1,4-dioxane. The reaction was heated in a microwave at 100 °C for 45 min under argon. After cooling to room temperature, the mixture was diluted with toluene and washed three times with brine. The organic layer was dried over MgSO_4_ and the solvent was removed by rotary evaporation to give a brown residue. The residue was purified with column chromatography (eluent DCM:MeOH; 19:1; *v*/*v*) to give a beige solid (41 mg, 73%).

**BC-BE**: ^1^H NMR (250 MHz, DMSO-*d*_6_): δ 9.24 (s, 1H), 8.18 (d, *J* = 6.7 Hz, 1H), 8.07 (t, *J* = 7.7 Hz, 2H), 7.93 (s, 1H), 7.72–7.53 (m, 1H), 7.47 (t, *J* = 7.5 Hz, 1H), 7.17 (d, *J* = 8.2Hz, 1H), 1.31 (s, 12H). TOF MS ES+ calcd for C_22_H_21_NO_4_SB 406.1284, found 406.1288. m.p. 229–233 °C.

### 3.4. Real-Time Monitoring of Oxidation **BC-BE** Boronic Probe in Cell-Free System Generating ^●^NO and O_2_^●–^

All solutions were prepared using deionized water (Millipore Milli-Q system). Due to poor solubility of probes in water, all experiments were performed with addition of an up 1% (*v*/*v*) of DMSO. The profiles of ONOO^–^ formation in a solution with hypoxanthine (HX) and xanthine oxidase (XO) as a source of steady flux of superoxide O_2_^●–^ and spermine-NONOate as a source of ^●^NO, were monitored using **BC-BA** as fluorogenic probe. The total fluorescence intensity of formed **BC-OH** dye was measured using a Beckman Coulter DTX880 plate reader. The instrument was kept at 37 °C during the measurements. The changes in fluorescence intensity were monitored over a 2 h period. ^●^NO fluxes were determined from the rate of decomposition of spermine-NONOate measured by following the decrease of its characteristic absorbance at 252 nm (ε = 8.5 × 10^3^ M^−1^ cm^−1^) [41]. The flux of O_2_^●–^ was determined by monitoring the cytochrome c(Fe^3+^) reduction and the increase in absorbance at 550 nm (using a difference in the values of the extinction coefficients between reduced and oxidized cytochrome of 2.1 × 10^4^ M^−1^ cm^−1^). In our experiments peroxynitrite was co-generated from fluxes of O_2_^●−^ and ^●^NO in the phosphate buffer (pH 7.4, 50 mM) with DTPA (100 µM), HX/XO (1 mM/70 µU/mL), spermine-NONOate (75 µM) and CAT (100 U/mL). Such systems produced up to 0.2 µM/min flux of peroxynitrite.

### 3.5. Cell Culture and Exposure Conditions

Human colon carcinoma cell line HT29 was obtained from ATCC, (Manassas, VA, USA). Cells were grown in DMEM with a 10% fetal bovine serum (FBS) medium supplemented with 100 U/mL penicillin, 100 µg/mL streptomycin, and 25 µg/mL amphotericin B. All cell culture experiments were performed in a humidified 5% CO_2_ and 95% atmosphere at 37 °C. All cell culture reagents were obtained from Life Technologies (Carlsbad, CA, USA).

### 3.6. Cell Metabolic Activity

Metabolic activity was evaluated with MTT assay. Briefly, cells were seeded into 96-well plate at 1 × 10^4^ cells/well density in 100 µL complete medium and grown overnight, and then incubated in the presence of studied compounds for another 8 h. After this, 20 µL of MTT reagent (5 mg/mL) was added for 120 min. After that time, MTT was removed, and formazan precipitates were solubilized by adding 100 µL of DMSO. Absorbance was measured at 570 nm using the Synergy 2 BioTek Microplate Reader (BioTek, Winooski, VT, USA).

## 4. Conclusions

We used 3-benzothiazol-2-yl-7-hydroxy-chromen-2-one fluorophore to develop a novel boronate probe for the selected biological oxidants. The **BC-BA** probe showed similar reactivity toward selected inflammatory oxidants to the previously reported **CBA** probe [17], producing fluorescent **BC-OH** phenolic product. In comparison to **CBA**, **BC-BA** is significantly more lipophilic, which may improve its cellular uptake. Furthermore, the fluorescence of the oxidation product is red shifted and its brightness is ca. 3-fold higher. This may help in the successful application of the probe for the imaging of biological oxidants in cultured cells. Detection of the minor products characteristic for a specific oxidant (ONOO^–^ or HOCl) will allow the unambiguous identification of the oxidants involved in probe oxidation.

Further structure modifications may be introduced to the developed scaffold to modulate the water solubility of the probe, fluorescence properties of the probe and the product and to target the probe to specific subcellular or extracellular compartments.

## Figures and Tables

**Table 1:** Photophysical properties of studied compounds.

	Solvent	λ_max_ [nm]	ε [10^4^ M^−1^ cm^−1^]	λ_ex_ [nm]	λ_em_ [nm]	Stokes Shift [nm]	Φ_em_	τ [ns] ^5^
**COH**	EtOH	326	1.22	326	391	65	0.10	
	H_2_O ^1^	323	1.27	323	452	129	0.80 ^3^ (0.76) ^4^	
**BC-OH**	EtOH	396	3.30	396	467	71	0.70 ^3^	3.07
	EtOH:PB ^2^	442	3.81	442	488	46	0.85 ^3^	3.32
**BC-H**	EtOH	364	2.36	364	464	100	0.14 ^3^	1.00
	EtOH:PB ^2^	365	2.72	365	470	105	0.09 ^3^	1.63
**BC-BA**	EtOH	369	2.22	369	464	95	0.21 ^3^	2.60
	EtOH:PB ^2^	371	2.55	371	473	102	0.17 ^3^	1.33

^1^ In H_2_O (pH 7.2) [20], ^2^ Aqueous phosphate buffer (100 mM, pH 7.4):EtOH (4:1), ^3^ According to quinine sulfate (Φ_ref_ 0.54 in water) [21], ^4^ NaHCO_3_–NaOH buffer (10 mM, pH 10.0, containing 1% EtOH) [35], ^5^ Fluorescence lifetime was determined by TCSPC single photon counting using FL900 spectrofluorometer(Edinburgh Instruments, Livingston, UK).

## Data Availability

The data presented in this study are available in article and supplementary material.

## Supplementary Materials

The following are available online. Figure S1: HPLC chromatograms of the BC-BA in aqueous solution containing phosphate buffer (0.1 M, pH 7.4), dtpa (10 µM) and EtOH (10%): freshly made solution (above), after 10 min (below). The traces were collected using the absorption detector set at 330 nm, Figure S2: ^1^H NMR spectrum of **BC-OH** in *d*_6_-DMSO, Figure S3: ^1^H NMR spectrum of **BC-H** in CDCl_3_, Figure S4: ^1^H NMR spectrum of **BC-OTf** in *d*_6_-DMSO, Figure S5: ^1^H NMR spectrum of **BC-BE** in *d*_6_-DMSO, Figure S6: Mass spectrum of **BC-OH**, Figure S7: Mass spectrum of **BC-H**, Figure S8: Mass spectrum of **BC-OTf**, Figure S9: Mass spectrum of **BC-BE**.

## Sample Availability

Samples of the compounds **BC-BA**, **BC-OH**, and **BC-H** are available from the authors.
