# Supplementary material for: Novel Boronate Probe Based on 3-Benzothiazol-2-yl-7-hydroxy-chromen-2-one for the Detection of Peroxynitrite and Hypochlorite
Source: Molecules. 2021 Sep 30;26(19):5940. doi: 10.3390/molecules26195940 (PMC8512868; doi:10.3390/molecules26195940)
Supplement: Supplementary file 1 [file molecules-26-05940-s001.zip › molecules-1380659-supplementary.pdf]

# Supplementary

# Novel boronate probe based on 3-benzothiazol-2-yl-7-hydroxy-chromen-2-one for detection of peroxynitrile and hypochlorite

Julia Modrzejewska,[1] Marcin Szala,[1] Aleksandra Grzelakowska,[1] Małgorzata Zakłos-Szyda,[2] Jacek Zielonka,[3*] Radosław Podsiadły[1][1]

[1] Institute of Polymer and Dye Technology, Faculty of Chemistry, Lodz University of Technology, Stefanowskiego 12/16, 90-924 Lodz, Poland; radoslaw.podsiadly@p.lodz.pl
[2] Faculty of Biotechnology and Food Sciences, Institute of Molecular and Industrial Biotechnology, Lodz University of Technology, Stefanowskiego 2/22, Łódź, 90-537, Poland
[3] Department of Biophysics, Medical College of Wisconsin, 8701 Watertown Plank Road, Milwaukee, WI 53226, United States; jzielonk@mcw.edu
* Correspondence: radoslaw.podsiadly@p.lodz.pl; +48 42 631-32-31 ; jzielonk@mcw.edu; (414) 955-4789

**Table of contents**

Hydrolysis of BC-BA .......... 2
$^{1}$H NMR Spectra .......... 3
Mass Spectra .......... 7

[1] Corresponding authors. E-mail addresses: julia.modrzejewska@dokt.p.lodz.pl, marcin.szala@p.lodz.pl, aleksandra.grzelakowska@p.lodz.pl, malgorzata.zaklos-szyda@p.lodz.pl, jzielonk@mcw.edu, radoslaw.podsiadly@p.lodz.pl

# Hydrolysis of BC-BA

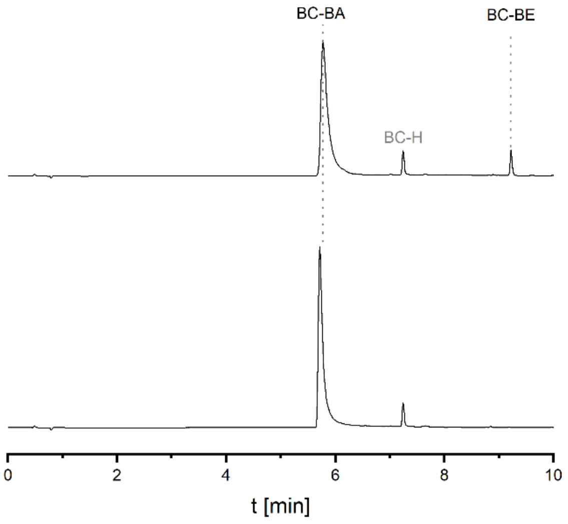


**Figure S1.** HPLC chromatograms of the BC-BA in aqueous solution containing phosphate buffer (0.1 M, pH 7.4), dtpa (10 μM) and EtOH (10%): freshly made solution (above), after 10 min (below). The traces were collected using the absorption detector set at 330 nm.

# $^{1}$H NMR Spectra

## BC-OH

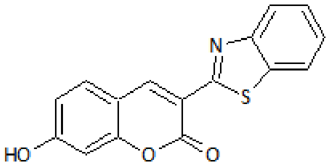


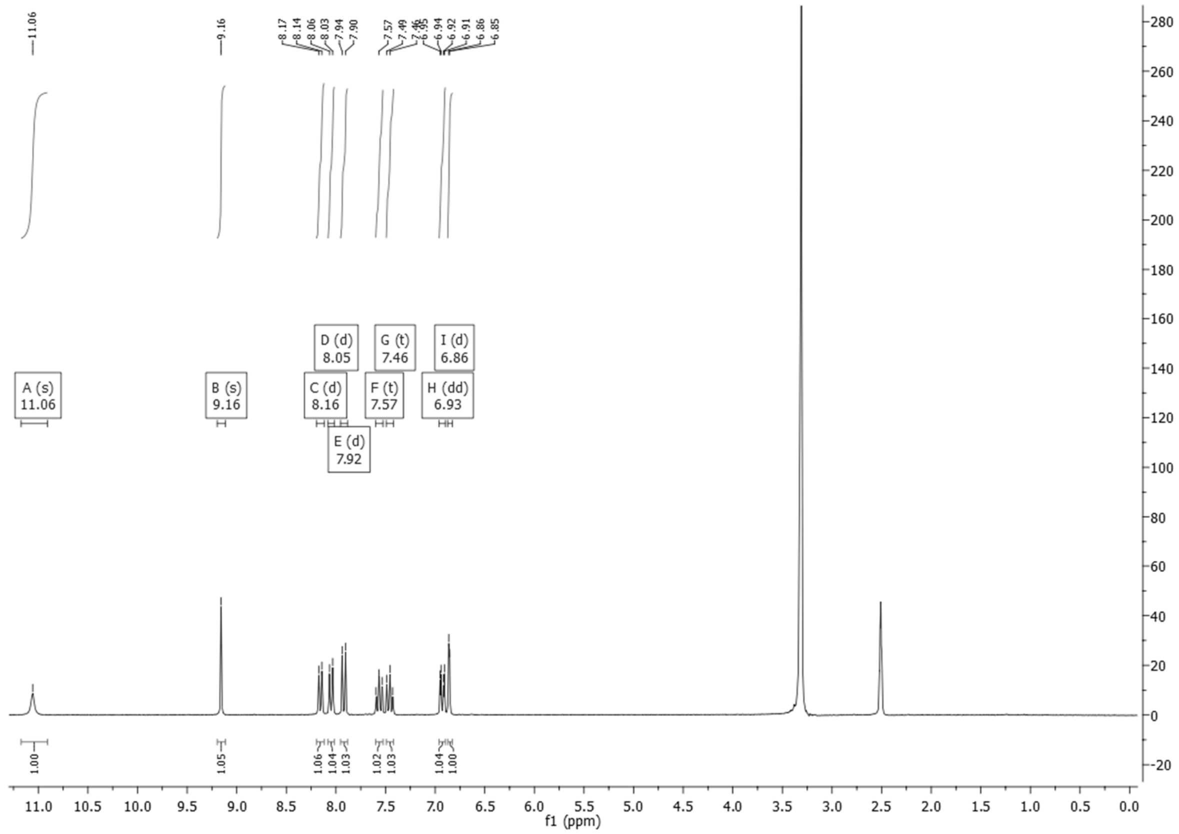


**Figure S2.** $^{1}$H NMR spectrum of **BC-OH** in DMSO-$d_6$.

# BC-H

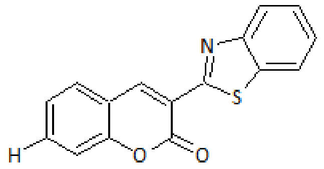


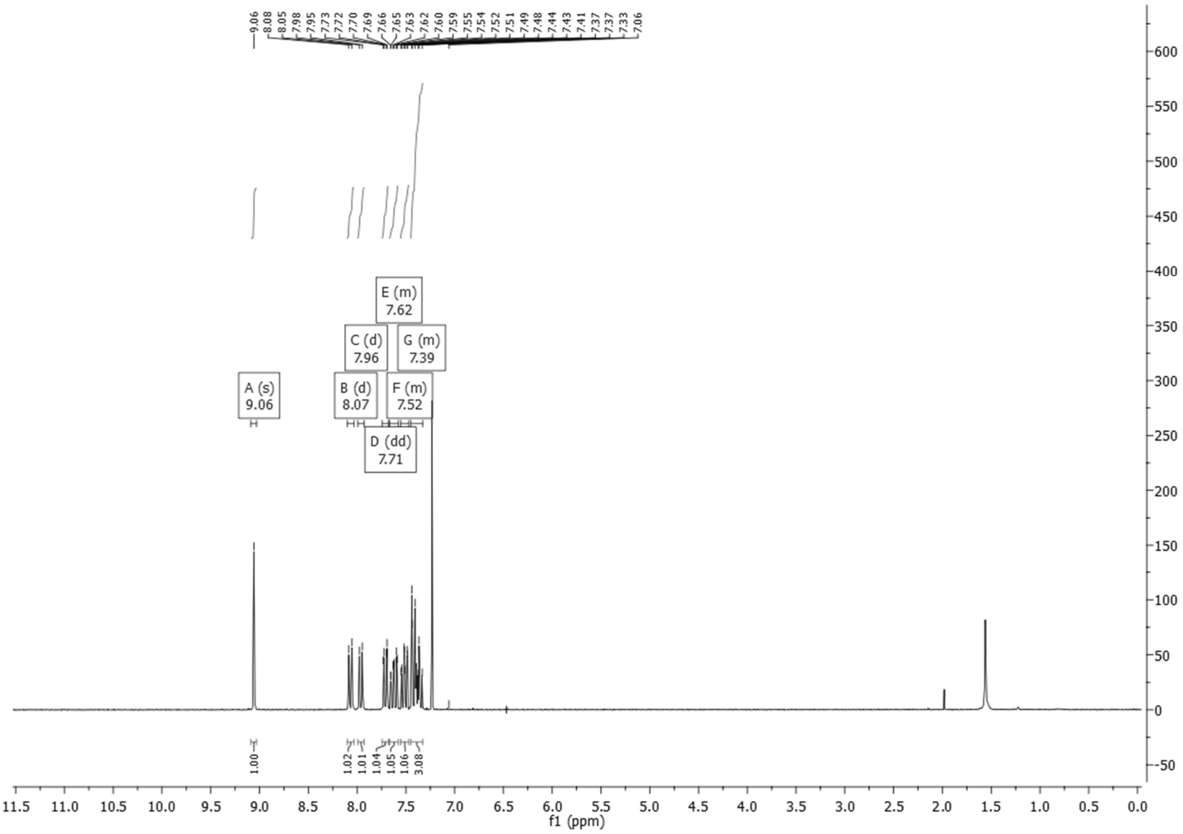


**Figure S3.** $^{1}$H NMR spectrum of **BC-H** in $CDCl_3$.

# BC-OTf

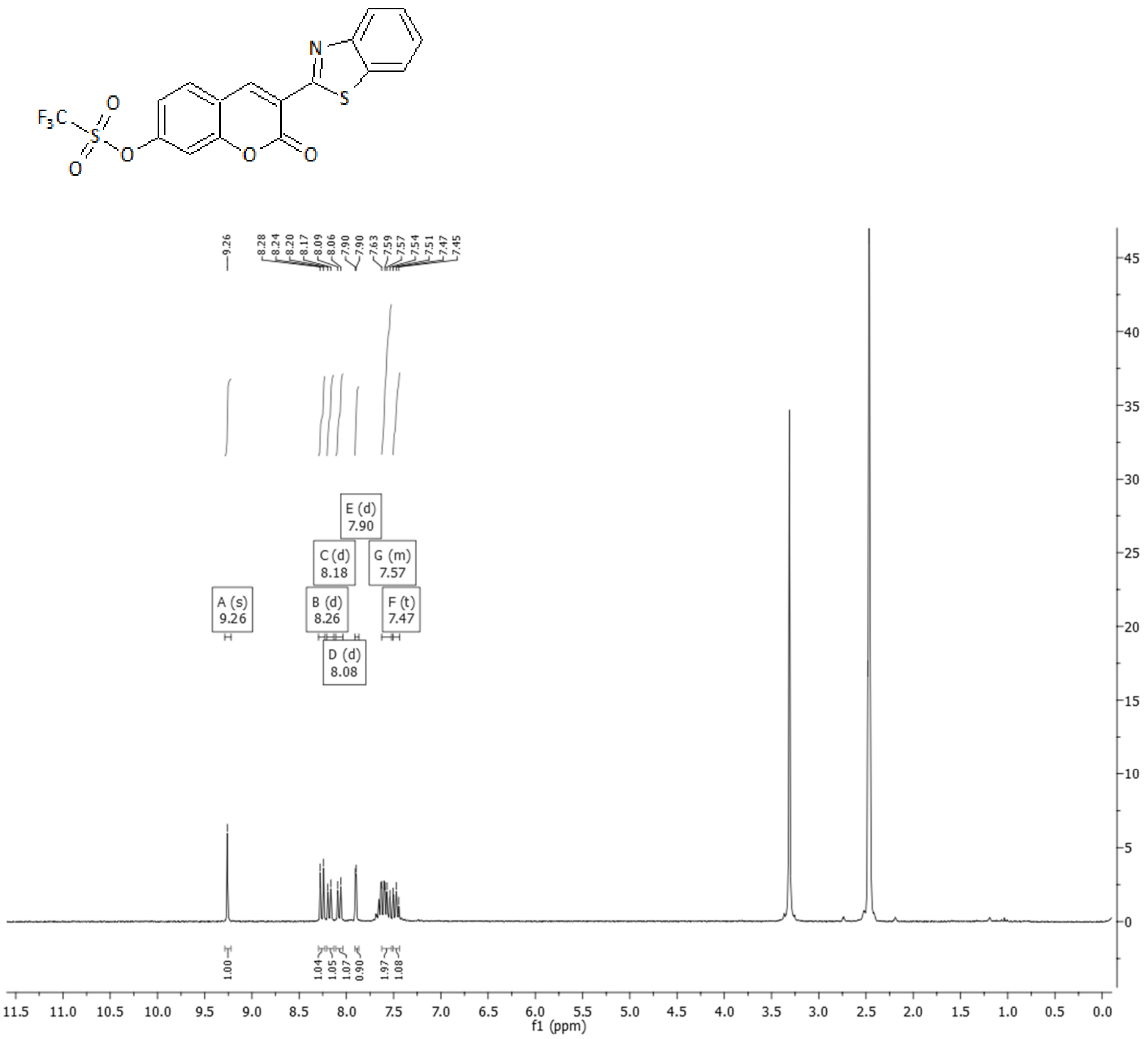


**Figure S4.** $^{1}$H NMR spectrum of **BC-OTf** in DMSO-$d_6$.

# BC-BE

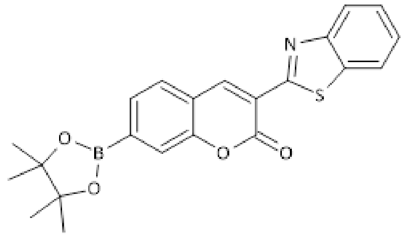


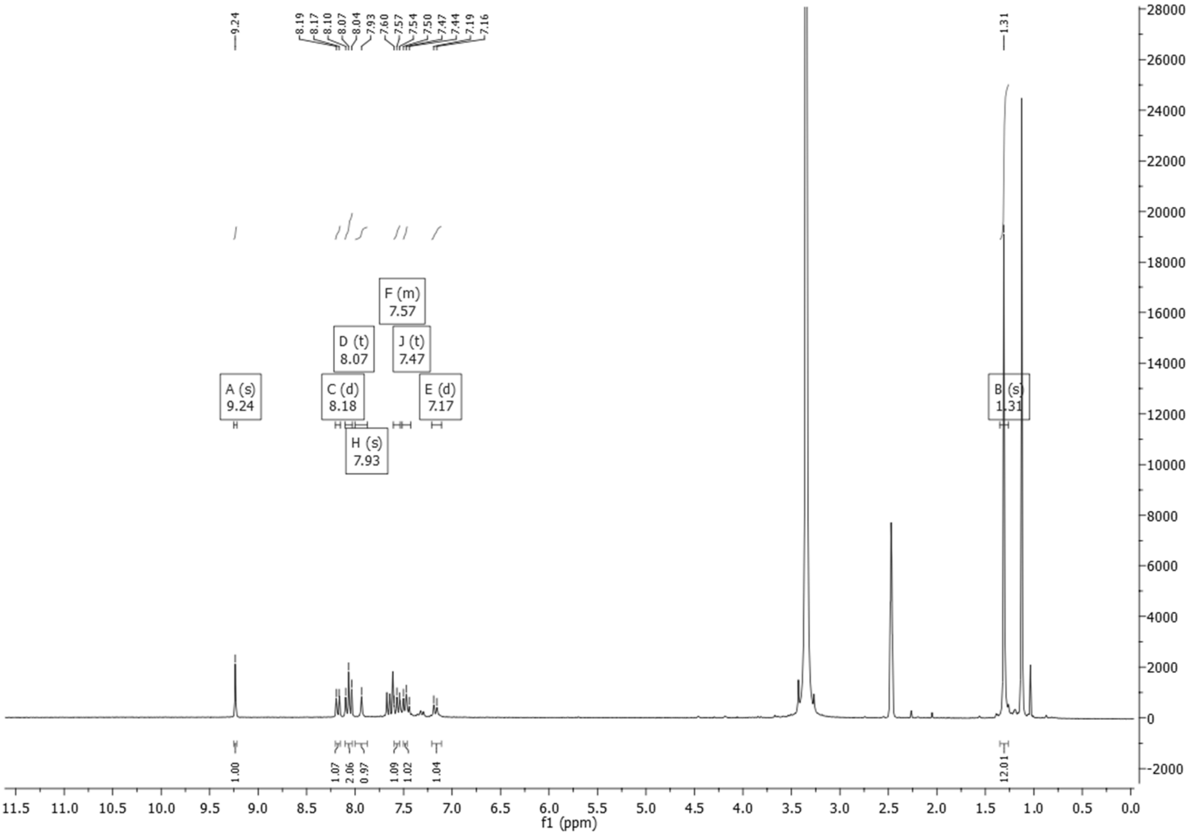


**Figure S5.** $^1$H NMR spectrum of **BC-BE** in DMSO-$d_6$.

# Mass Spectra

## BC-OH

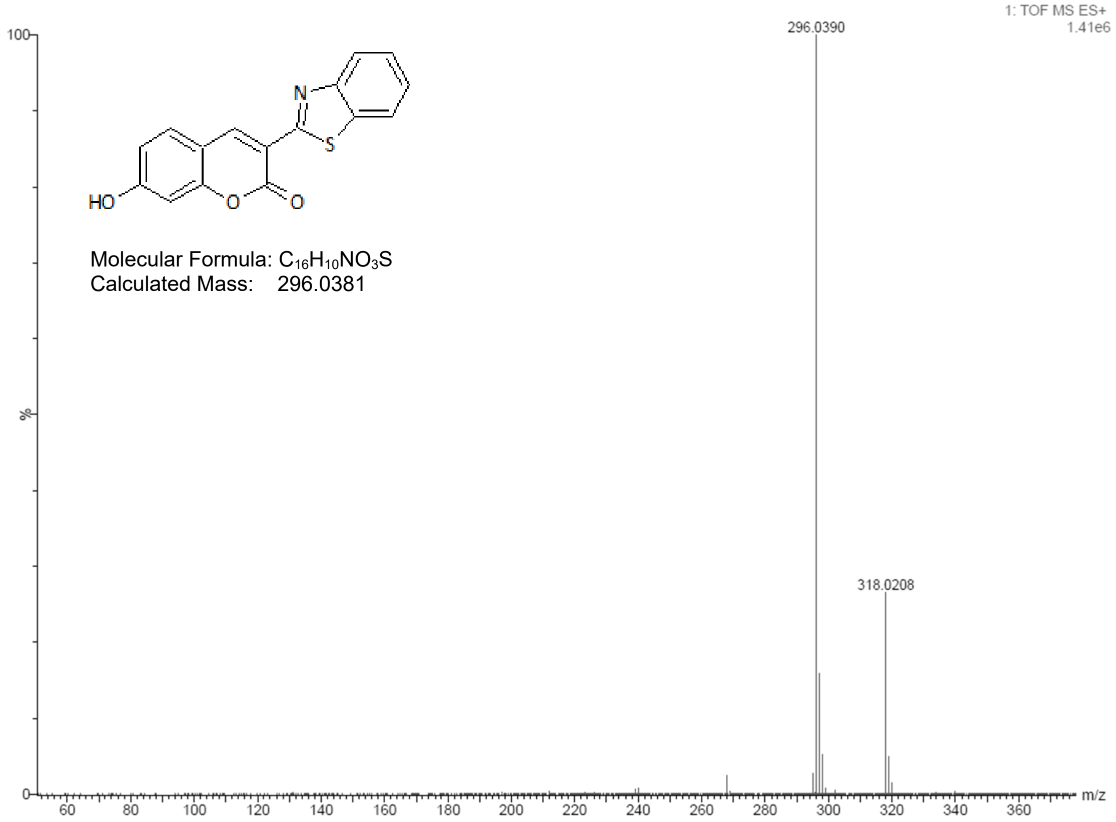


**Figure S6.** Mass spectrum of **BC-OH**.

# BC-H

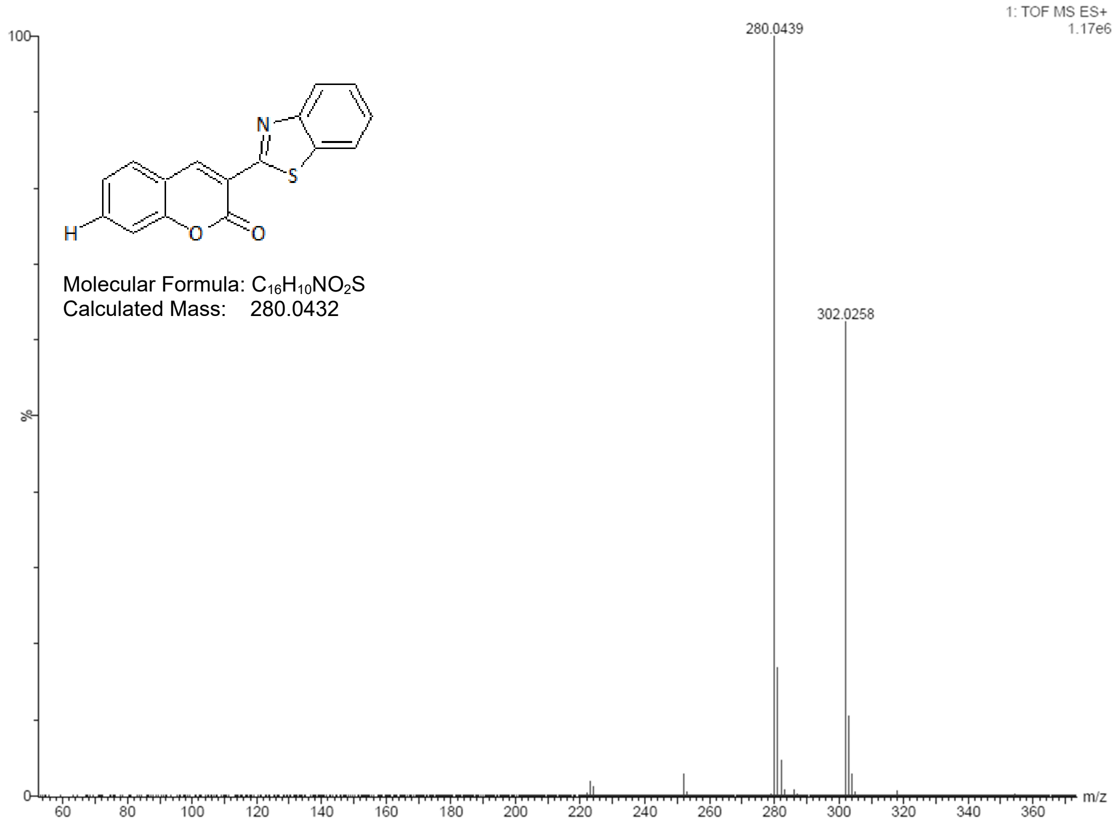


**Figure S7.** Mass spectrum of **BC-H**.

# BC-OTf

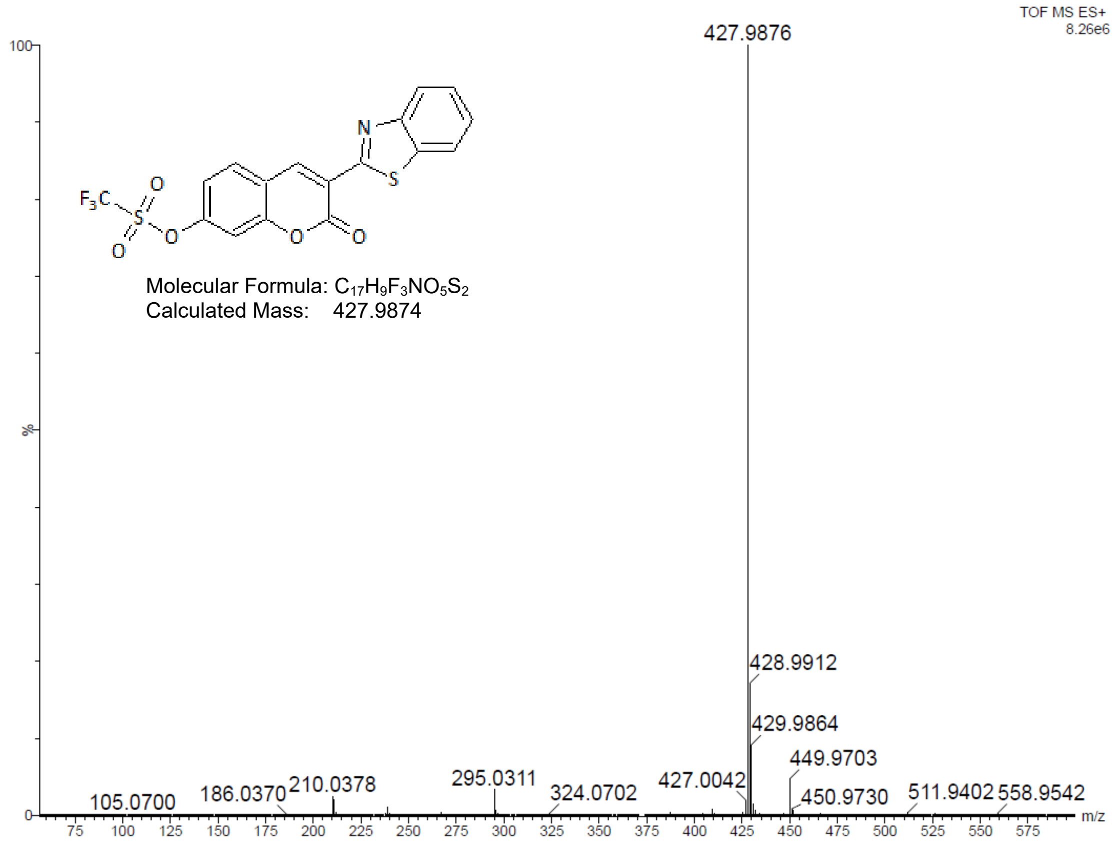


**Figure S8.** Mass spectrum of **BC-OTf**.

# BC-BE

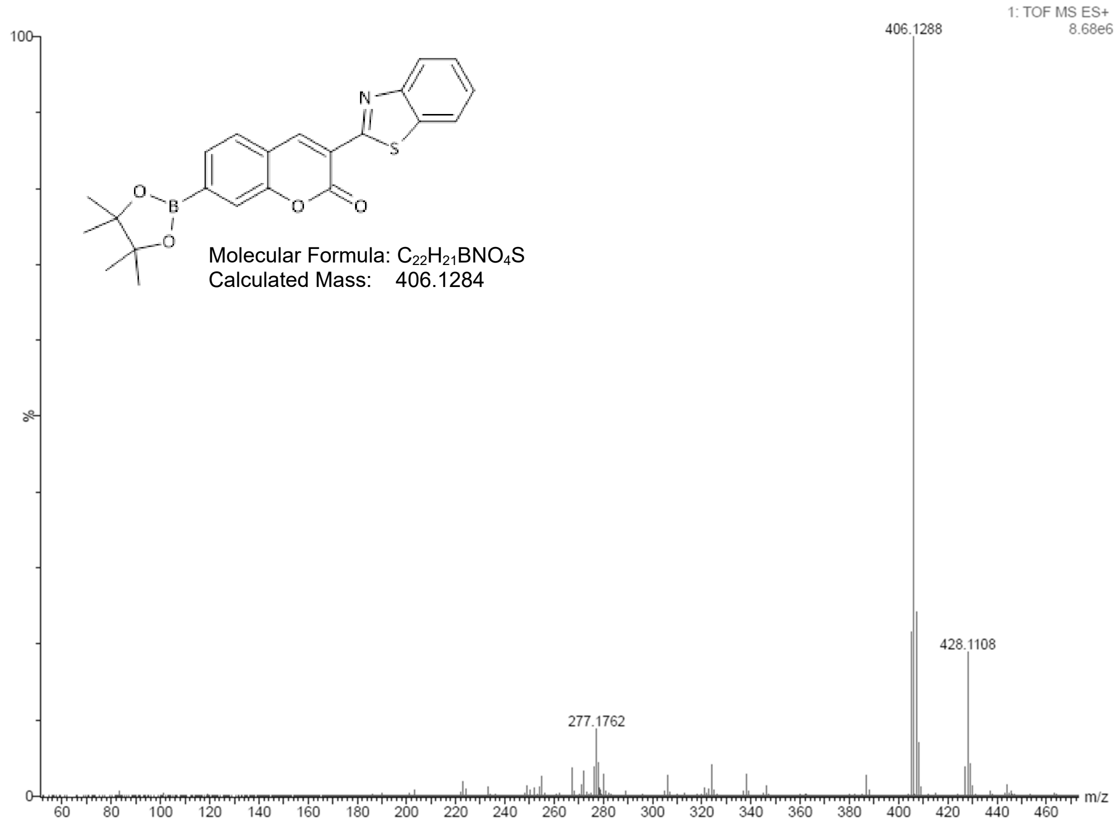


**Figure S9.** Mass spectrum of **BC-BE**.
